# Expression and Role of E-Cadherin, β-Catenin, and Vimentin in Human Papillomavirus–Positive and Human Papillomavirus–Negative Oropharyngeal Squamous Cell Carcinoma

**DOI:** 10.1369/0022155420950841

**Published:** 2020-08-14

**Authors:** Hesham Mohamed, Caj Haglund, Lauri Jouhi, Timo Atula, Jaana Hagström, Antti Mäkitie

**Affiliations:** Department of Pathology, University of Helsinki and Helsinki University Hospital, Helsinki, Finland; Department of Histology, Omar Al-Mukhtar University, Al-Bayda, Libya; Department of Surgery, University of Helsinki and Helsinki University Hospital, Helsinki, Finland; Research Program Unit, Translational Cancer Biology, University of Helsinki, Helsinki, Finland; Department of Otorhinolaryngology—Head and Neck Surgery, University of Helsinki and Helsinki University Hospital, Helsinki, Finland; Department of Otorhinolaryngology—Head and Neck Surgery, University of Helsinki and Helsinki University Hospital, Helsinki, Finland; Department of Pathology, University of Helsinki and Helsinki University Hospital, Helsinki, Finland; Research Program Unit, Translational Cancer Biology, University of Helsinki, Helsinki, Finland; Department of Oral Pathology and Radiology, University of Turku, Turku, Finland; Department of Otorhinolaryngology—Head and Neck Surgery, University of Helsinki and Helsinki University Hospital, Helsinki, Finland; Research Program in Systems Oncology, Faculty of Medicine, University of Helsinki, Helsinki, Finland; Division of Ear, Nose and Throat Diseases, Department of Clinical Sciences, Intervention and Technology, Karolinska Institutet, Karolinska University Hospital, Stockholm, Sweden

**Keywords:** cancer, immunohistochemistry, p16, tissue microarray

## Abstract

Oropharyngeal squamous cell carcinoma (OPSCC) is subclassified by the World Health Organization into two different entities: human papillomavirus (HPV)-positive and HPV-negative tumors. HPV infection promotes the epithelial-to-mesenchymal transition (EMT) and transformation of keratinocyte stem cells into cancer stem cells. EMT is a crucial process in the carcinogenesis of epithelial-derived malignancies, and we aimed to study the role of its markers in OPSCC. This study consists of 202 consecutive OPSCC patients diagnosed and treated with curative intent. We examined E-cadherin, β-catenin, and vimentin expression using immunohistochemistry and compared these with tumor and patient characteristics and treatment outcome. We found that the cell-membranous expression of β-catenin was stronger in HPV-positive than in HPV-negative tumors, and it was stronger in the presence of regional metastasis. The stromal vimentin expression was stronger among HPV-positive tumors. A high E-cadherin expression was associated with tumor grade. No relationship between these markers and survival emerged. In conclusion, β-catenin and vimentin seem to play different roles in OPSCC: the former in the tumor tissue itself, and the latter in the tumor stroma. HPV infection may exploit the β-catenin and vimentin pathways in carcinogenic process. More, β-catenin may serve as a marker for the occurrence of regional metastasis:

## Introduction

Epithelial-to-mesenchymal transition (EMT) plays an important role in the carcinogenesis of epithelial-derived tumors. During this process, the epithelial cells lose their cell polarity and cell-to-cell adhesion, and acquire invasive and migratory properties. These cells express cancer stem cell markers and are, therefore, named cancer stem cells (CSCs).^1,2^ CSC markers are expressed in various cancers, including oral and oropharyngeal cancers, and their expression may be valuable in predicting clinical outcome.^3,4^ Recently, human papillomavirus (HPV) was shown to promote the transformation of keratinocyte stem cells to become CSCs, possibly explaining the high metastasis rate of HPV-related oropharyngeal tumors.^5^ The p16 gene is a well-known tumor suppressor protein, encoded by a gene localized on chromosome 9p21.^6^ Protein p16 expression highly correlates with the presence of HPV16 in oropharyngeal squamous cell carcinoma (OPSCC),^7,8^ and patients with HPV-positive OPSCC exhibit better survival compared with their virus-negative counterparts.^8,9^

The cadherin family is a group of glycoproteins regulating the Ca^2+^-dependent cell-to-cell adhesion mechanism. Takeichi^10^ has identified E-cadherin as the first member of the cadherin family. It is a tumor suppressor,^11^ and its low expression is a hallmark for EMT.^12^ In addition, a low E-cadherin expression is a characteristic of many types of cancers^3,13,14^ and typically a marker of poor prognosis.^15,16^ Catenins (α, β, and γ) are cytoplasmic proteins, first described by Ozawa et al.^17^ These proteins connect E-cadherin to actin filaments in adherent junctions^18^ and are involved in signal transduction via cell adhesion,^17^ particularly β-catenin,^19^ which is tightly associated with E-cadherin.^18^ In addition to the aforementioned functions of the β-catenin protein, the β-catenin gene is also known as an oncogene located on chromosome 3p21, a region involved in cancer development.^20^ Furthermore, the β-catenin protein may be involved in cancer pathogenesis through the Wnt/β-catenin pathway.^21–23^ Vimentin is a protein normally expressed in mesenchymal cells.^24,25^ In normal epithelial tissues, vimentin is involved in cell adhesion through its interaction with vinculin and integrin.^26,27^ Again, it may play a role in the adhesion and transcellular migration of lymphocytes through the endothelial cells.^28^ In addition, vimentin may affect DNA transcription and cell apoptosis by interacting with the transcriptional determinant of p53.^29^ Moreover, vimentin is a canonical marker of EMT, and its positivity serves as a valuable marker for metastasis occurrence in epithelial-derived malignancies.^30,31^

Here, we studied the expression of vimentin, E-cadherin, and β-catenin in OPSCC. We compared their expression levels with clinical parameters and outcomes, and examined the relationship between these markers and HPV status.

**Figure 1.**
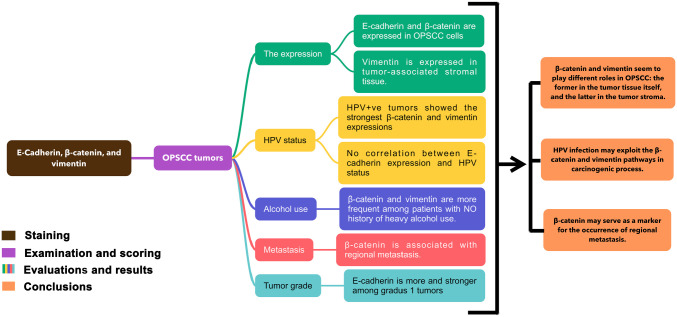
Graphical abstract describing the workflow in this study: immunohistochemistry staining, evaluation of the scoring results with clinical and pathological data, results, and conclusions. Abbreviations: HPV, human papillomavirus; OPSCC, oropharyngeal squamous cell carcinoma.

Figure 1 describes the workflow in which the main procedures, results, and conclusions are described in this publication.

## Materials and Methods

### Patient Selection

Altogether, 331 patients with oropharyngeal cancer were diagnosed and treated at the Department of Otorhinolaryngology—Head and Neck Surgery, Helsinki University Hospital (Helsinki, Finland), between January 1, 2000, and December 31, 2009. Of these patients, the following were excluded from the study: those receiving palliative treatment (*n*=44), those with concurrent (*n*=5) or previously treated head and neck squamous cell carcinoma (HNSCC) (*n*=11), a histology other than squamous cell carcinoma (SCC) or an SCC subtype (*n*=18), or those for whom tumor tissue was unavailable (*n*=51 for E-cadherin and β-catenin, and *n*=52 for vimentin). Thus, our study cohort comprised 202 patients for E-cadherin and β-catenin and 201 for vimentin expression analysis.

### Hospital Records Data Source

We recorded clinicopathological data from patient files. The median follow-up time for patients was 5 years, but all patients had a minimum follow-up of 3 years or until death. We obtained the dates and causes of death from Statistics Finland. The patient data are described in detail in our previous publication.^32^ The Research Ethics Board of the Hospital District of Helsinki and Uusimaa approved the study design, and an institutional study permission was granted.

### Immunohistochemistry

We obtained formalin-fixed and paraffin-embedded surgical tissue samples from the archives of the Department of Pathology. The slides were re-evaluated by an experienced head and neck pathologist (J.H.), and cancer areas were marked on the slides. We prepared tissue microarray (TMA) blocks from the donor paraffin blocks. From the selected cancer areas, four tumor spots were detached for each case by a 1-mm needle and placed into a recipient paraffin block with a semiautomatic tissue microarrayer (Beecher Instruments; Silver Spring, MD).^33^ From the TMA blocks, 4-μm-thick sections were cut, deparaffinized in xylene, and rehydrated through a graded alcohol series. We accomplished antigen retrieval by heating the samples in 98C EDTA buffer (pH 9.0) for 24 min in a pretreatment PT Module (Lab Vision Corp.; Fremont, CA). The samples were then cooled to room temperature for 1 hr. Endogenous peroxidase was inactivated by incubating the specimens in Dako REAL Peroxidase-Blocking Solution for 5 min. We used a specific primary antibody for each marker: mouse anti-human p16^INK4a^ monoclonal antibody (MAb) incubated for 30 min (9517 CINtec Histology Kit; mtm laboratories, Heidelberg, Germany), mouse anti-β-catenin MAb diluted to 1:400 and incubated for 30 min (CAT-5H10, 18-0226; Invitrogen Corporation, Carlsbad, CA; www.invitrogen.com), mouse anti-E-cadherin MAb diluted to 1:200 and incubated for 30 min (HECD-1, 13-1700; Invitrogen Corporation), and mouse anti-vimentin MAb diluted to 1:4000 and incubated for 1 hr (V9, M0725; Dako; Santa Clara, CA; https://www.citeab.com/antibodies/2414896-m0725-vimentin-concentrate). Staining was achieved using the Dako Real EnVision Detection System and peroxidase DAB+ with an Autostainer (LabVision; Fremont, CA).

For all markers, we used colon cancer tissue as the positive control, except for p16, for which the positive control was tongue squamous cell carcinoma tissue. In each staining, the negative control was a slide without the primary antibody.

### HPV In Situ Hybridization and p16 Immunostaining

HPV in situ hybridization (ISH) and p16 immunohistochemical staining were performed previously by our group.^32^ We have achieved the Ventana Inform HPV ISH assay using a high-risk HPV probe (16, 18, 31, 33, 35, 39, 45, 51, 52, 56, 58, and 66) and the iVIEW Blue detection kit in Benchmark XT series stainer (Ventana Medical Systems; Tuscon, AZ). In this study, only HPV+/p16+ tumors were considered HPV-related OPSCC.

### Scoring

Two independent investigators (H.M. and J.H.) evaluated the immunopositivity in tumor cells in a blinded manner without knowledge of the clinicopathological data. In the case of any disparity, an accordant score was used for further analysis. We scored both the cytoplasmic and cell-membranous expression of β-catenin, and the cell-membranous expression of E-cadherin and the cytoplasmic expression of vimentin according to the intensity. For β-catenin and E-cadherin, the scoring was as follows: negative (0), weakly positive (1), moderately positive (2), or strongly positive (3).^34,35^ Vimentin was scored as negative (0), weak (1), and strong (2).^36^ For all markers, the highest score for the four spots from each tumor was used for further analysis.

### Statistical Analysis

We used SPSS version 20.0 (SPSS, Inc.; Chicago, IL) to analyze all data. The scoring results for different markers were compared with clinical and pathological data. We used the Chi-square test to analyze the material with asymptotic and exact *p* values when most suitable, except when examining the relationships between the markers and patients’ age, which were analyzed using ANOVA. The Kaplan–Meier estimate was used to calculate the 5-year disease-specific survival (DSS) rate and recurrence-free survival (RFS) rate, using the log-rank statistical test. We considered value of *p*<0.05 as statistically significant.

## Results

### Expression of Markers

**Figure 2.**
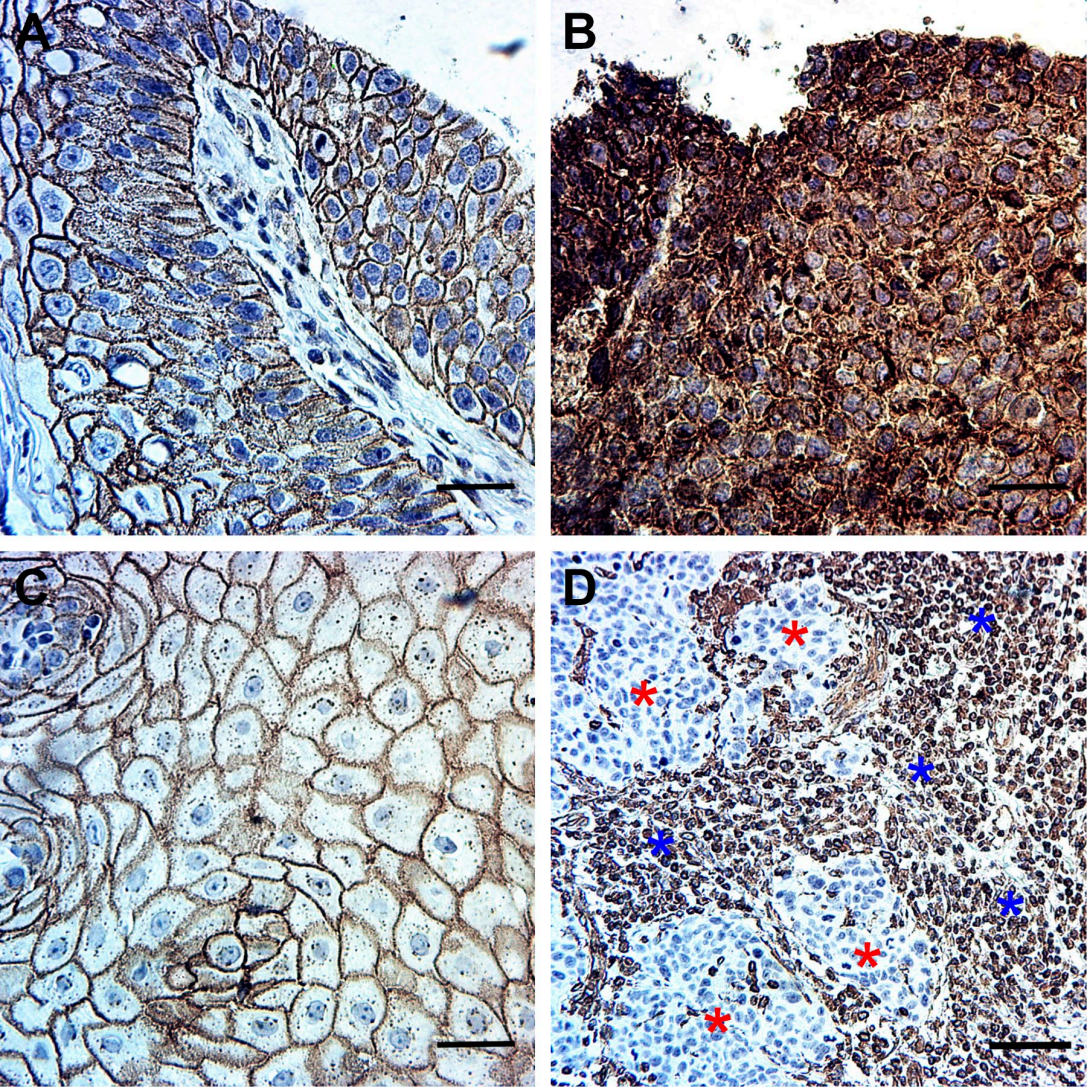
Immunohistochemical staining pattern of β-catenin, E-cadherin, and vimentin in oropharyngeal carcinoma (OPSCC). (A) The cell-membranous expression of β-catenin in OPSCC (magnification, 40×). (B) The cytoplasmic expression of β-catenin in OPSCC (magnification, 40×). (C) The cell-membranous expression of E-cadherin (magnification, 40×). (D) Vimentin expression in the tumor-related stromal tissue of OPSCC is denoted by “blue stars,” whereas the tumor tissue remained negative, which is denoted by “red stars” (magnification, 20×). Scale bar: 50 µm. Abbreviation: OPSCC, oropharyngeal squamous cell carcinoma.

**Figure 3.**
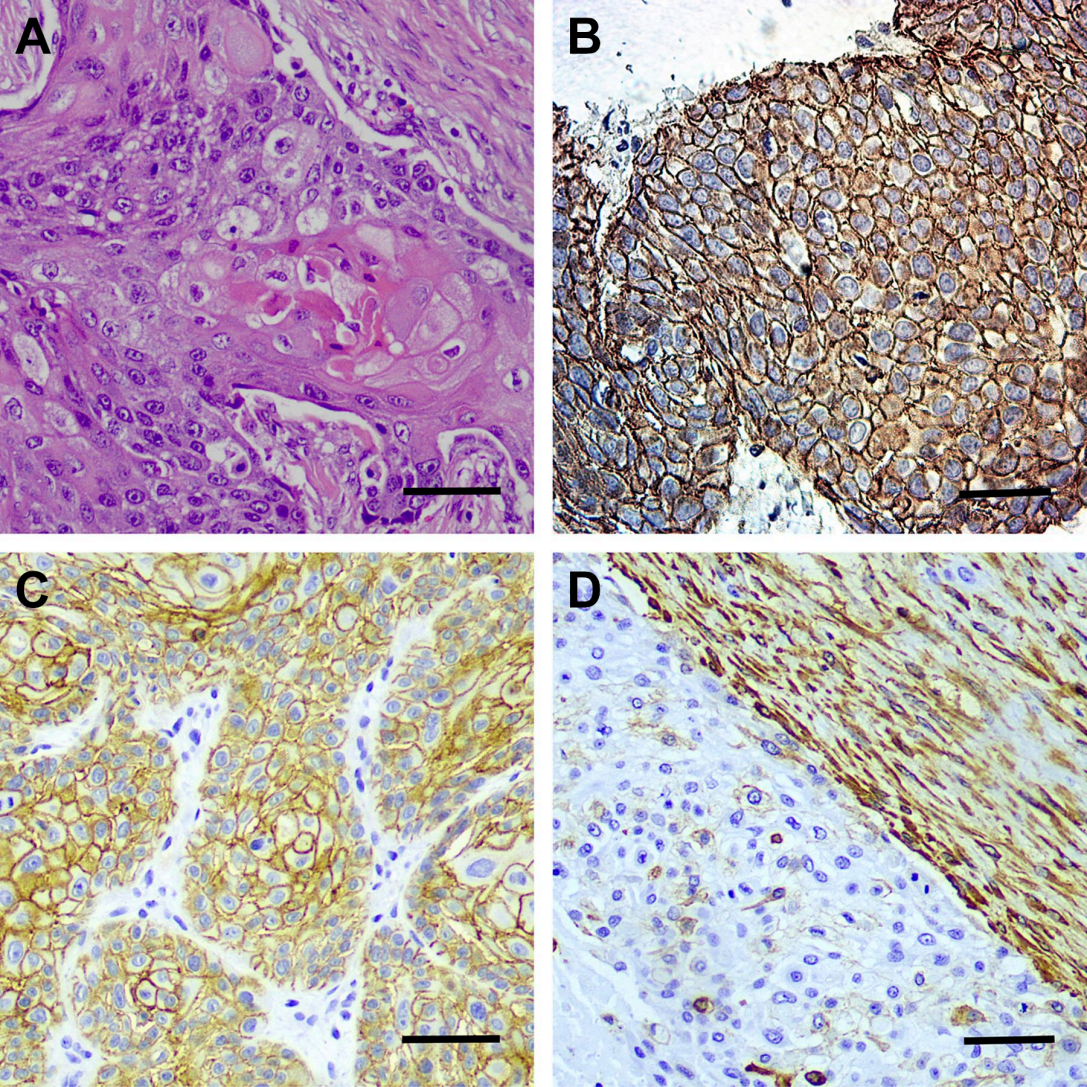
A tumor sample with different staining. (A) H&E staining (magnification, 200×). (B) The cell-membranous and cytoplasmic expression of β-catenin in OPSCC (magnification, 200×). (C) The cell-membranous expression of E-cadherin (magnification, 40×). (D) Vimentin expression (magnification, 200×). Scale bar: 50 µm. Abbreviation: OPSCC, oropharyngeal squamous cell carcinoma.

The cell-membranous expression of E-cadherin and both the cytoplasmic and cell-membranous expression of β-catenin were present in the tumor cells, whereas vimentin was expressed in the tumor-related stromal tissue cells. In the tumor cells, we found no vimentin positivity (Figs. 2 and 3).

### Relationship Between E-Cadherin, β-Catenin, and Vimentin Expression

E-cadherin was positively associated with both the cell-membranous and cytoplasmic expression of β-catenin (Table 1). In addition, vimentin expression was associated with cell-membranous β-catenin, although we found no significant relationship between the vimentin and E-cadherin expression (Table 2).

**Table 1.** Expression of E-Cadherin and Its Association With Clinicopathological Factors in OPSCC.

Variables	E-Cadherin Scoring					*p* Value
	Negative	Weak	Moderate	Strong	Total Number	
Sex						
Men	8	23	73	46	150	
Women	2	7	24	19	52	
Total	10	30	97	65	202	0.414*
Smoking						
Never	1	3	14	8	26	
Ex-smoker	2	12	22	13	49	
Regularly	5	12	48	31	96	
Total	8	27	84	52	171	0.798**
Heavy drinking						
No	1	13	28	19	61	
Previously	1	3	15	5	24	
Yes	1	4	19	14	38	
Total	3	20	62	38	123	0.414**
HPV						
Positive	4	16	48	33	101	
Negative	6	14	49	32	101	
Total	10	30	97	65	202	0.796*
Grade						
Gr1	0	1	8	9	18	
Gr2	2	7	48	21	78	
Gr3	8	22	41	35	106	
Total	10	30	97	65	202	0.020*
T class						
T1	3	5	19	11	38	
T2	2	11	40	23	76	
T3	1	7	21	17	46	
T4	4	7	17	14	42	
Total	10	30	97	65	202	0.944*
N class						
N0	1	4	21	13	39	
N+	9	26	76	52	163	
Total	10	30	97	65	202	0.370*
Stage						
I–II	0	1	19	10	30	
III–IV	10	29	78	55	172	
Total	10	30	97	65	202	0.101*
Tumor site						
Anterior wall	3	9	25	24	61	
Lateral wall	6	19	56	36	117	
Posterior wall	0	0	1	2	3	
Superior wall	1	2	15	3	21	
Total	10	30	97	65	202	0.449**
Cell-membranous β-catenin expression						
Negative	2	4	5	1	12	
Weak	3	5	15	8	31	
Moderate	4	10	41	15	70	
Strong	1	11	36	41	89	
Total	10	30	97	65	202	<0.001**
Cytoplasmic β-catenin expression						
Negative	7	10	17	9	43	
Weak	2	2	12	4	20	
Moderate	1	8	31	16	56	
Strong	0	10	37	36	83	
Total	10	30	97	65	202	<0.001**

Abbreviations: HPV, human papillomavirus; OPSCC, oropharyngeal squamous cell carcinoma. Boldfaced values are statistically significant for those *P* < 0.05 and statistically highly significant for those *P* < 0.001.

* Chi-square test with asymptotic *p*-value. **Chi-square test with exact *p*-value.

**Table 2.** Expression of Vimentin and its Association With Clinicopathological Factors in OPSCC.

Variables	Vimentin Scoring				
	Negative	Weak	Strong	Total Number	*p* Value
Sex					
Men	1	83	65	149	
Women	0	26	26	52	
Total	1	109	91	201	0.429
Smoking					
Never	0	11	15	26	
Ex-smoker	0	24	25	49	
Regularly	1	53	42	96	
Total	1	88	82	171	0.158
Heavy drinking					
No	0	29	32	61	
Previously	1	12	11	24	
Yes	0	28	10	38	
Total	1	69	53	123	**0.016**
HPV					
Positive	1	45	54	100	
Negative	0	64	37	101	
Total	1	109	91	201	**0.026**
Grade					
Gr1	0	9	9	18	
Gr2	0	42	36	78	
Gr3	1	58	46	105	
Total	1	109	91	201	0.595
T class					
T1	0	21	16	37	
T2	1	45	30	76	
T3	0	20	26	46	
T4	0	23	19	42	
Total	1	109	91	201	0.414
N class					
N0	0	20	19	39	
N+	1	89	72	162	
Total	1	109	91	201	0.603
Stage					
I–II	0	14	16	30	
III–IV	1	95	75	171	
Total	1	109	91	201	0.336
Tumor site					
Anterior wall	0	37	24	61	
Lateral wall	1	58	57	116	
Posterior wall	0	2	1	3	
Superior wall	0	12	9	21	
Total	1	109	91	201	0.747
Cell-membranous β-catenin expression					
Negative	0	7	4	11	
Weak	0	21	10	31	
Moderate	0	41	20	70	
Strong	1	40	48	89	
Total	1	109	91	201	**0.026**
Cytoplasmic β-catenin expression					
Negative	0	24	18	42	
Weak	0	5	15	20	
Moderate	0	39	17	56	
Strong	1	41	41	83	
Total	1	109	91	201	0.447
E-cadherin					
Negative	0	6	3	9	
Weak	0	19	11	30	
Moderate	1	48	48	97	
Strong	0	36	29	65	
Total	1	109	91	201	0.251

Abbreviations: HPV, human papillomavirus; OPSCC, oropharyngeal squamous cell carcinoma.

Chi-square test was done with exact *p*-value.

### Relationship Between the Markers and HPV Status

#### E-Cadherin

E-cadherin expression was almost identical in both HPV-positive and HPV-negative tumors (96% and 94%, respectively). E-cadherin expression did not correlate with HPV status (*p*=0.796) (Table 1).

#### β-Catenin

The cell-membranous expression of β-catenin appeared in 97% (98/101) of HPV-positive tumors and in 91% (92/101) of HPV-negative tumors. The cell-membranous expression of β-catenin was stronger in HPV-positive than in HPV-negative tumors (*p*=0.001) (Table 3).

**Table 3.** Cell Membranous and Cytoplasmic Expression of β-Catenin and Their Associations With Clinicopathological Factors in OPSCC.

Variables	Cell-Membranous β-Catenin Scoring						Cytoplasmic β-Catenin Scoring					
	Negative	Weak	Moderate	Strong	Total Number	*p* Value	Negative	Weak	Moderate	Strong	Total Number	*p* Value
Sex												
Men	11	20	54	65	150		34	16	37	63	150	
Women	1	11	16	24	52		9	4	19	20	52	
Total	12	31	66	87	202	0.687*	38	21	55	82	202	0.588*
Smoking												
Never	1	4	7	14	26		3	5	7	11	26	
Ex-smoker	2	6	18	23	49		13	6	17	13	49	
Regularly	7	15	34	40	96		18	6	24	48	96	
Total	10	25	55	75	171	0.243*	34	17	48	72	171	0.307*
Heavy drinking												
No	2	5	23	31	61		8	7	20	26	61	
Previously	1	2	10	11	24		6	2	7	9	24	
Yes	5	7	12	14	38		6	3	9	20	38	
Total	8	14	45	56	123	0.020**	20	12	36	55	123	0.810*
HPV												
Positive	3	14	26	58	101		20	13	27	41	101	
Negative	9	17	44	31	101		23	7	29	42	101	
Total	12	31	70	89	202	0.001*	43	20	56	83	202	0.952*
Grade												
Gr1	1	1	5	11	18		3	1	5	9	18	
Gr2	6	17	30	25	78		13	8	24	33	78	
Gr3	5	13	35	53	106		27	11	27	41	106	
Total	12	31	70	89	202	0.387*	43	20	56	83	202	0.138*
T class												
T1	2	6	13	17	38		11	1	11	15	38	
T2	2	16	25	33	76		11	10	20	35	76	
T3	3	3	20	20	46		6	5	15	20	46	
T4	5	6	12	19	42		15	4	10	13	42	
Total	12	31	70	89	202	0.674*	43	20	56	83	202	0.224*
N class												
N0	2	13	11	13	39		5	7	14	13	39	
N+	10	18	59	76	163		38	13	42	70	163	
Total	12	31	70	89	202	0.036*	43	20	56	83	202	0.946*
Stage							2	6	10	12	30	
I–II	2	9	8	11	30		41	14	46	71	172	
III–IV	10	22	62	78	172		43	20	56	83	202	0.357*
Total	12	31	70	89	202	0.121*						
Tumor site												
Anterior wall	3	10	20	28	61		14	7	14	26	61	
Lateral wall	8	15	39	55	117		27	11	34	45	117	
Posterior wall	0	0	2	1	3		0	0	1	2	3	
Superior wall	1	6	9	5	21	0.199**	2	2	7	10	21	0.118**
Total	12	31	70	89	202		43	20	56	83	202	

Abbreviations: HPV, human papillomavirus; OPSCC, oropharyngeal squamous cell carcinoma.

* Chi-square test with asymptotic *p*-value. **Chi-square test with exact *p*-value.

Cytoplasmic β-catenin expression was identified in 81% of HPV-positive and in 77% of HPV-negative tumors. We found no correlation between the cytoplasmic expression of β-catenin and HPV status (*p*=0.952) (Table 3).

#### Vimentin

Vimentin expression was negative in OPSCC cells in all tumor samples. We observed vimentin immunoexpression in tumor-associated stromal tissue in 99% of HPV-positive and 100% of HPV-negative tumors. In addition, 54% (54/100) of HPV-positive tumors exhibited a strong vimentin expression, whereas only 37% (37/101) of HPV-negative tumors exhibited a strong vimentin expression. The stromal vimentin expression was stronger among HPV-positive tumors (*p*=0.026) (Table 2).

### Correlation Between Markers and Other Clinicopathological Parameters and Survival

#### Alcohol Use

Both the vimentin and cell-membranous expression of β-catenin appeared more frequently among patients with no history of heavy alcohol use (*p*=0.020 and *p*=0.016, respectively) (Tables 2 and 3).

#### Metastases

Moderate or strong expression was seen in 83% (135/163) of HPV-positive tumors and only in 62% (24/39) of HPV-negative tumors. The cell-membranous expression of β-catenin was positively associated with the presence of regional metastasis (*p*=0.036) (Table 3).

#### Tumor Grade

E-cadherin was significantly associated with tumor grade. It was expressed in all grade 1 tumor samples, but not in all grade 2 and 3 tumors. In addition, 50% of grade 1, 27% of grade 2, and 33% of grade 3 tumors exhibited a strong E-cadherin expression (*p*=0.020) (Table 1).

#### Other Clinicopathological Factors

We found no significant relationship between β-catenin (cell-membranous and cytoplasmic), E-cadherin, and vimentin expression and gender, smoking, tumor class (T class), or tumor stage and patient age (Tables 1–4).

**Table 4.** The Relationship Between Age and the Prognostic Markers Used in the Study (E-Cadherin, β-Catenin, and Vimentin) With ANOVA.

	Sum of Squares	Difference of Freedom	Mean Square	*F* Ratio	*p* Value
E-cadherin					
Between groups	72.849	3	24.283	0.240	0.869
Within groups	20,053.412	198	101.280		
Total	20,126.261	201			
Cell-membranous β-catenin					
Between groups	104.310	3	34.770	0.344	0.794
Within groups	20,021.951	198	101.121		
Total	20,126.261	201			
Cytoplasmic β-catenin					
Between groups	211.451	3	70.484	0.701	0.553
Within groups	19,914.810	198	100.580		
Total	20,126.261	201			
Vimentin					
Between groups	289.437	2	144.719	1.450	0.237
Within groups	19,757.510	198	99.785		
Total	20,046.947	200			

#### Survival

We found no significant relationship between the examined markers and patient survival.

## Discussion

In this study, the cell-membranous expression of β-catenin was stronger in HPV-positive tumors than in HPV-negative tumors. This result is in accordance with the result of a previous study showing that β-catenin may accelerate HPV-mediated cervical carcinogenesis through the Wnt/β-catenin pathway.^21^ Thus, β-catenin may play the same role in HPV-related OPSCC. In addition, we found that a strong vimentin expression was more frequently seen in HPV-positive tumors than in HPV-negative tumors. We detected β-catenin positivity in OPSCC cells, whereas vimentin expression was solely seen in OPSCC-related stromal tissue and not in tumor cells. Schäfer et al.^37^ have suggested that vimentin may act as a binding protein for HPV16 pseudovirion and modulate the internalization of HPV16 pseudovirion in cervix carcinoma cell lines; with the results being adverse compared with ours, both HPV-positive and HPV-negative tumor cells were negative for vimentin expression, and we found no relation between stromal vimentin expression and HPV status.

In our article, E-cadherin expression was associated with both cell-membranous and cytoplasmic β-catenin immunoexpression. Similarly, this association has been identified in previous studies on different types of cancers.^38,39^ In addition, we found an association between the high expression of β-catenin and the presence of regional lymph node metastasis, a relationship that has been earlier identified in breast cancer too.^22^ Furthermore, in OPSCC, the low E-cadherin expression was associated with an increased risk of distant metastasis.^40^ Another study on breast cancer showed that the normal expression pattern of all proteins composing cadherin–catenin complex was associated with the absence of metastasis, and the alteration in the expression of one of these proteins impairs the adhesion and antimetastatic functions of other proteins in the same complex, increasing the risk of metastases.^41^ In our study, we observed a relationship between the presence of regional lymph node metastasis and a high expression of β-catenin. Thus, the high expression of β-catenin may hinder the cell-to-cell adhesion function of E-cadherin, increasing the risk of invasion and metastases, although we found no significant relationship between E-cadherin expression and metastasis.

Heavy alcohol use appears to associate with the development of HPV-negative OPSCC, and even light drinking appears to increase the risk of developing OPSCC.^42^ A study on hepatocellular carcinoma showed that alcohol consumption invigorates the Wnt/β-catenin signaling pathway, stimulating hepatocyte proliferation and promoting carcinogenesis.^43^ In addition, a study by Christopher et al.^44^ found that alcohol stimulates the expression of vimentin in breast and colon cancer cells. Contrary to this finding, we found that β-catenin and vimentin expression more frequently appeared among patients with no history of heavy alcohol use. One explanation for this discrepancy may be that different cancers have different biology and activate distinct signaling pathways.

In oral cancer, low E-cadherin expression appears to associate with a poor prognosis.^16^ In addition, high-grade OPSCC has a high recurrence rate.^45^ In HPV-negative OPSCC, García-Pedrero et al.^46^ showed that low membranous β-catenin and E-cadherin expression was associated with a poorer overall survival. In our cohort, a low E-cadherin expression was associated with a high tumor grade, although we found no correlation with prognosis. Similar results were previously found in studies of oral, oropharyngeal, and penile cancers.^14,16,47^ Although we found no relationship between E-cadherin and patient survival, we can deduce that the patients with low E-cadherin expression typically exhibit high-grade tumors, which have a higher likelihood of recurrence, and this may impact the survival.

To conclude, β-catenin, E-cadherin, and vimentin appear to have different roles in the pathogenesis of OPSCC. In addition, β-catenin may serve as a marker for the occurrence of regional metastasis. Moreover, in HPV-positive OPSCC, HPV infection may exploit the β-catenin and vimentin pathways in the carcinogenesis process—the former in the cancer tissue itself, and the latter in the tumor stroma.
